# 

**Published:** 1867-11

**Authors:** 


### CHICAGO

### MEDICAL JOURNAL.

VOL. XXIV. NOVEMBER, 1867. NO. 11.

CHICAGO:
ROBERT FERGUS' SONS, PRINTERS,
12 & 14 CLARK STREET.
1867.

